# Inhibition of Venezuelan Equine Encephalitis Virus Using Small Interfering RNAs

**DOI:** 10.3390/v14081628

**Published:** 2022-07-26

**Authors:** Amrita Haikerwal, Michael D. Barrera, Nishank Bhalla, Weidong Zhou, Niloufar Boghdeh, Carol Anderson, Farhang Alem, Aarthi Narayanan

**Affiliations:** 1 Center for Infectious Disease Research, George Mason University, Manassas, VA 20110, USA; ahaikerw@gmu.edu (A.H.); mbarrer@gmu.edu (M.D.B.); nbhalla@gmu.edu (N.B.); nboghdeh@gmu.edu (N.B.); cander47@gmu.edu (C.A.); falem@gmu.edu (F.A.); 2 Center for Applied Proteomics and Molecular Medicine, George Mason University, Manassas, VA 20110, USA; wzhou@gmu.edu

**Keywords:** Venezuelan Equine Encephalitis Virus, nonstructural protein 2, host proteome, mass spectrometry, viral proteome, RNA interference, siRNA therapeutics, Argonaute 2, RISC complex

## Abstract

Acutely infectious new world alphaviruses such as Venezuelan Equine Encephalitis Virus (VEEV) pose important challenges to the human population due to a lack of effective therapeutic intervention strategies. Small interfering RNAs that can selectively target the viral genome (vsiRNAs) has been observed to offer survival advantages in several in vitro and in vivo models of acute virus infections, including alphaviruses such as Chikungunya virus and filoviruses such as Ebola virus. In this study, novel vsiRNAs that targeted conserved regions in the nonstructural and structural genes of the VEEV genome were designed and evaluated for antiviral activity in mammalian cells in the context of VEEV infection. The data demonstrate that vsiRNAs were able to effectively decrease the infectious virus titer at earlier time points post infection in the context of the attenuated TC-83 strain and the virulent Trinidad Donkey strain, while the inhibition was overcome at later time points. Depletion of Argonaute 2 protein (Ago2), the catalytic component of the RISC complex, negated the inhibitory effect of the vsiRNAs, underscoring the involvement of the siRNA pathway in the inhibition process. Depletion of the RNAi pathway proteins Dicer, MOV10, TRBP2 and Matrin 3 decreased viral load in infected cells, alluding to an impact of the RNAi pathway in the establishment of a productive infection. Additional studies focused on rational combinations of effective vsiRNAs and delivery strategies to confer better in vivo bioavailability and distribution to key target tissues such as the brain can provide effective solutions to treat encephalitic diseases resulting from alphavirus infections.

## 1. Introduction

New world encephalitic alphaviruses, including Venezuelan Equine Encephalitis Virus (VEEV) are classified as category B select agents by the National Institutes of Health (NIH) and the Centers for Disease Control and Prevention (CDC). VEEV is a positive-strand RNA virus belonging to the family *Togaviridae*, characterized by an 11.4 Kb genome [1,2]. The genome encodes for four nonstructural proteins (nsP1, 2, 3 and 4) and five structural proteins (Capsid, 6K, E1, E2 and E3). Disease following VEEV infection occurs naturally in humans in many parts of the world due to transmission by infected mosquitoes [2]. Infections have been recorded for several decades in the Americas, primarily associated with natural transmission [3,4,5]. One of the largest natural VEEV outbreaks was reported in Columbia in 1972, which resulted in 23,283 human infections, 960 cases of neurological manifestations and 156 deaths [4]. VEEV is highly infectious in an aerosol form and is known to cause encephalitic disease due to infection of the central nervous system [6]. There are no FDA-approved vaccines, therapeutics or prophylactics available to protect from or treat encephalitic disease due to VEEV exposure. TC-83, a live attenuated strain of VEEV, and C-84, a formalin inactivated preparation based on TC-83, are used to vaccinate at risk personnel [7,8]. However, while TC-83 induces robust seroconversion, immunoreactivity concerns persist, with several vaccinees reporting experiencing disease-like symptoms. C-84 is a poor immunogen without robust seroconversion and is used as a booster if required.

There continues to be significant emphasis on host–pathogen interactions for acutely infectious viruses towards the goal of identifying host-based therapeutic candidates. Such studies have been conducted in the context of the Ebola virus outbreak and the recent SARS-CoV-2 pandemic and potential host-based intervention strategies have been identified [9,10,11,12,13]. Interactome studies involving VEEV nonstructural and structural proteins also underscore this idea [14,15]. Deciphering host–virus interactomes can identify critical vulnerabilities in the viral multiplication cycle that may be targeted by small molecules to achieve inhibition, without leading to the development of resistance. Understanding the host–virus interactome will also be informative in deciphering the nature of innate immune mechanisms that are relevant for pathogen control and may be susceptible to modulation by the pathogen.

RNA interference (RNAi) is an evolutionarily conserved innate immune mechanism that is dependent on cytoplasmic dsRNA that silences gene expression in a sequence-specific manner at both transcriptional and post-transcriptional level [16]. Plant, invertebrates, nematodes and fungi utilize the RNAi pathway as an antiviral strategy by recognizing viral dsRNA intermediates [17]. The viral dsRNA is processed by Dicer, an RNase III nuclease, to generate virus-specific small interfering RNAs (vsiRNAs) of approximately 21–23 nucleotides. Dicer and transactivation response element RNA-binding protein (TRBP) facilitate the loading of one strand of the duplex vsiRNA into the RNA-induced silencing complex (RISC), which contains the Argonaute effector nuclease. The vsiRNA strand acts as a guide RNA to direct RISC to the complementary RNA. The degree of complementarity between the guide strand and target (complementary) strand determines whether RNA silencing is achieved by site-specific cleavage of the target RNA or by translational repression of the target RNA molecule. Plant and arthropod viruses are shown to have proteins that antagonize synthesis of vsiRNA and inhibit the RNAi pathway [18,19,20,21,22]. However, the dynamics of interactions between viral proteins and the host RNAi pathway as mediators of proviral or antiviral responses remain poorly understood.

In the current study, we explored the utility of vsiRNAs directed against the VEEV genome to achieve inhibition of the virus in cell culture. Our data demonstrate that transfected vsiRNAs can achieve robust inhibition of the TC-83 and the TrD strains of VEEV in infected cells. Our observations support the idea that modulation of the RNAi pathway by VEEV may contribute significantly to the ability of VEEV to establish a productive infection in mammalian cells.

## 2. Materials and Methods

### 2.1. Cell Culture and Viruses

293T human embryonic kidney cells (ATCC, CRL-3216) and Vero African green monkey kidney cells (ATCC, CCL-81) were obtained from the American Type Culture Collection (Manassas, VA, USA). 293T and Vero cells were cultured at 37 °C and 5% CO_2_ in Dulbecco’s Modified Eagle’s Medium (DMEM, 112-013-101CS, Quality Biological, Gaithersburg, MD, USA) supplemented with 4.5 g/L glucose, 2 mM l-glutamine (MT2005CI, FisherSci, Chicago, IL, USA), 10% heat-inactivated fetal bovine serum (FBS, 10437028, ThermoFisher, Carlsbad, CA, USA) for 293T cells or 5% heat-inactivated fetal bovine essence (FBE, 10805-184, VWR, Dixon, CA, USA) for Vero cells with 10 µg/mL streptomycin and 10 U/mL penicillin (45000-652, VWR).

The live-attenuated strain TC-83 strain of VEEV (Accession: L01443) and the wild-type, virulent Trinidad Donkey (TrD) VEEV strain (Accession: J04332) were obtained from BEI Resources (NR-332).

### 2.2. siRNA Design and Synthesis

Double-stranded siRNAs specific to the TC-83 and TrD strains (20 in total) were designed and synthesized at siDESIGN Center, Horizon Discovery Ltd. (Boulder, CO, USA). The individual siRNAs with their targeted nucleotide sequence of the sense strands are listed in Table 1. The selected anti-VEEV siRNA sequences were verified to be uniquely represented in the VEEV genome and absent in human and mouse genomes. The designed siRNAs were synthesized using ON-TARGETplus technology.

Five nanomoles of ON-TARGETplus siRNAs of human MATR3 or MATRN3 (Dharmacon, L-017382-00-0005), DICER1 (Dharmacon, L-003483-00-0005), TARBP2 or TRBP2 (Dharmacon, L-017430-00-0005), Ago2 or EIF2C2 (Dharmacon, D-004639-03-0020), MOV10 (L-014162-00-0005) were reconstituted to 10 µM in molecular-grade water. ON-TARGETplus nonspecific siRNA control (Dharmacon, D-001810-10-05) was used as a negative control. All siRNAs were reconstituted according to the manufacturer’s instructions prior to use.

### 2.3. Transfection

293T cells were transfected with anti-VEEV siRNAs, anti-RISC complex siRNAs (Ago2, MATRN3, DICER1, MOV10 and TRBP2 siRNA) or with the negative control siRNAs. Briefly, 293T cells were seeded at 10^5^ cells/well in 24-well plates or 2 × 10^5^ cells/well in 12-well plates coated with poly-l-lysine (0.1 mg/mL) 24 h prior to transfection. The final concentration of siRNA was maintained at 20 nM for transfection, unless otherwise mentioned. siRNAs were transfected using DharmaFECT1 Transfection Reagent (Horizon Discovery Ltd., T-2001-02) according to manufacturer’s guidelines.

### 2.4. Infection

293T cells were infected at a multiplicity of infection (MOI) 0.1 or 0.01 following published methods [14,15]. The virus was diluted in complete DMEM culture media to obtain the indicated MOI. At the time of infection, the viral inoculum was added to the monolayer and the cells were maintained for 1 h at 37 °C. After 1 h, the viral inoculum was removed, complete DMEM culture media was added back to the monolayer and the infected cells were maintained at 37 °C and 5% CO_2_. At different time points post infection, viral supernatants were collected and stored at −80 °C prior to being subjected to quantification assays.

### 2.5. Cell Viability

293T cells were seeded in 24-well white-wall plates at a seeding density of 10^5^ cells/well. Following transfection, cell viability was measured using CellTiterGlo^®^ Luminescent Cell Viability Assay according to the manufacturer’s instructions (Promega, G7572, Madison, WI, USA). Luminescence was measured using a Beckman Coulter DTX 880 multimode plate reader and data recorded as relative light units (RLU).

### 2.6. Enzyme-Linked Immunosorbent Assay (ELISA)

Interferon-β protein expression level was measured using a VeriKine Human IFN Beta ELISA Kit (pbl Assay Science (Piscataway, NJ, USA), 41410) following manufacturer’s instructions. Briefly, samples were diluted in sample diluent, incubated for 1 h, treated with Antibody solution for 1 h after which HRP solution was added to each well. Samples were then incubated with TMB substrate solution and incubated at room temperature in the dark for 15 min. After the addition of Stop solution, absorbance was determined at 450 nm using a microplate reader.

### 2.7. Immunoprecipitation

Two milligrams of total protein were incubated overnight at 4 °C with rotation using 2 µg mouse IgG3 isotype control (Abcam, 18394, Cambridge, UK) or anti-VEEV nsP2 antibody (KeraFast, EU015) or anti-MATRN3 (Novus, NB100-1761). Magnetic Dynabeads coated with protein G (FisherSci, 10-003-D) were washed with citrate phosphate buffer pH 5.0 (50 mM Tris-HCL pH 7.5, 120 mM NaCl, 5 mM EDTA, 0.5% NP-40, 50 mM NaF, 0.2 mM Na_3_VO_4_, Protease cocktail tablet) (Sigma-Aldrich, 11697498001, St. Louis, MO, USA) and added to the protein–antibody IP complexes. Rotation at room temperature proceeded for 40 min followed by 1× wash with TNE100 + 0.1% NP-40, 1 wash with TNE50 + 0.1% NP-40, and 2 wash with PBS. TNE buffers consisted of 100 mM Tris-HCl pH 7.5 and 0.2 mM EDTA, with 100 mM NaCl for TNE100 or 50 mM NaCl for TNE50. For Western blot imaging, Laemmli buffer supplemented with 100 mM DTT was added and beads were boiled for 10 min. For samples subjected to mass spectrometry, the last PBS wash was removed and Dynabeads were stored at 80 °C until processed for analysis.

### 2.8. Western Blot

Whole cell lysates were prepared from infected or transfected 293T cells. Briefly, the cell culture medium was removed, and cells were lysed using 1× clear lysis buffer (CLB, Cell Signaling Technology, 9803, Danvers, MA, USA) and were supplemented with 1 mM phenylmethylsulphonyl fluoride (PMSF, Cell Signaling Technology, 8553S). Cell lysates were centrifuged at 10,000 rpm for 10 min at 4 °C and supernatants were collected in a separate microcentrifuge tube. Protein concentrations were measured using the standard curve of 1 mg/mL bovine serum albumin (BSA, Fisher Scientific, BP1600, Hampton, NH, USA) with Bradford reagent (VWR, E530-1L). Equal volumes of protein samples were diluted 1:1 in 2× Laemmli buffer (1610737, Bio-Rad, Hercules, CA, USA) followed by boiling for 10 min. Protein samples were electrophoresed on 4–20% Tris-Glycine Gels (ThermoFisher, XP04122) and transferred to polyvinyl difluoride (PVDF) membranes by wet transfer in 4 °C for 2 h at 250 mA. PVDF membranes were blocked at room temperature with 3% nonfat dry milk in Tris-buffered saline and 0.1% Tween-20 (TBS-T) for 30 min. Primary antibodies such as anti-Ago2 (Abcam, ab186733), anti-DICER1 (Abcam, ab264250), anti-MATRN3 (Novus, NB100-1761), anti-MOV10 (Abcam, ab176687) and anti-TRBP2 (Abcam, ab180947) were diluted in 3% BSA in TBS-T at 1:1000 dilution and incubated on individual membranes overnight at 4 °C. PVDF membranes were washed thrice for 10 min and incubated with respective secondary HRP-conjugated antibody (FisherSci, PI32460) diluted in 3% nonfat dry milk in TBS-T at 1:10,000 dilution for 1 h at room temperature. Then, membranes were quickly washed thrice with TBST for 5 min and thrice with TBS for 5 min. Membranes were then imaged with SuperSignal West Femto Maximum Sensitivity Substrate Kit (ThermoFisher, 34095) using a Bio-Rad Molecular Imager ChemiDoc XRS system. To quantify actin expression as a loading control for all the samples, membranes were reprobed with a mild stripping buffer consisting of 0.1 M glycine, 0.2 M NaCl, 0.1% Tween-20 at pH 2.5, followed by blocking (3% BSA in TBS-T for 30 min), incubation with HRP-conjugated anti-actin antibody (Abcam, ab49900), diluted in 3% BSA, TBS-T for 30 min and washing with TBS-T thrice. Membranes were imaged with the ChemiDoc XRS system (Biorad, Hercules, CA, USA). NIH ImageJ software calculated band densities and signals were normalized to actin.

### 2.9. Liquid Chromatography–Mass Spectrometry

LC–MS/MS was performed as previously described [12]. Briefly, immunoprecipitated Dynabeads were treated with 8 M urea and disulfide bonds were reduced with 1 M DTT and alkylated with iodacetamide. Following a trypsin digestion (ThermoFisher, 25200056) for 4 h at 37 °C, peptides were eluted with ZipTip purification (Millipore, Z720070, Rockville, MD, USA), and resuspended in H_2_O with 0.1% formic acid. Orbitrap Fusion tandem MS/MS with nanospray reverse-phase liquid chromatography (ThermoFisher) was performed. Full-scan mass spectra were acquired in Orbitrap over 300 to 2000 *m*/*z* with a 30,000 resolution followed with MSn scans by CID activation mode. The 3 most intense ions were selected for fragmentation using a 35 collision energy and activation at Q = 25 for 30 milliseconds. Dynamic exclusion and charge state rejection were enabled. Mass spectra were fitted against NCBI reference sequence AAB02516 or P27282 for the nonstructural polyprotein sequence analysis with Sequest Bioworks software 3.3.1 (ThermoFisher).

### 2.10. Plaque Assay 

Two hundred thousand (2 × 10^5^) Vero cells/well were seeded in a 12-well plate and incubated for 24 h. Viral supernatants were serially diluted in culture media and the dilutions were overlaid on cells for viral attachment for 1 h; 0.6% agarose was diluted with Eagle’s Minimum Essential Medium (without phenol red, supplemented with 5% fetal bovine essence, nonessential amino acids, 1 mM sodium pyruvate (45000-710, VWR), 2 mM l-glutamine, 20 U/mL penicillin, and 20 g/mL streptomycin) in 1:1 ratio. Forty-eight hours post infection, the monolayer was fixed for 1 h with 10% paraformaldehyde (F79P-4, FisherSci). Agar plugs were removed, and cells were stained with 1% crystal violet (FisherSci, C581-25) in a 20% ethanol solution (BP2818-4, FisherSci). Plaques were counted and recorded.

### 2.11. Statistical Analysis

Statistical analyses were carried out using Graph Pad Prism 7. Statistical significance was determined using one-way ANOVA with Dunnett’s post test unless otherwise stated. Statistical significance values are indicated using asterisks: * *p* < 0.0332, ** *p* < 0.0021, *** *p* < 0.0002, **** *p* < 0.0001, and ns for not significant.

## 3. Results

### 3.1. Design of vsiRNAs That Target the VEEV Genome

Our goal was to develop vsiRNAs that can effectively target the VEEV genome and achieve inhibition of VEEV in cell culture. To that end, we designed twenty vsiRNAs that targeted conserved regions in the nonstructural and structural genes in the Trinidad Donkey (TrD) and the TC-83 strains. The vsiRNA sequences, target locations in the VEEV genome and the corresponding genes are indicated in Table 1. 

### 3.2. Efficacy of Anti-VEEV siRNAs in Reducing TC-83 Replication In Vitro

**Figure 1:**
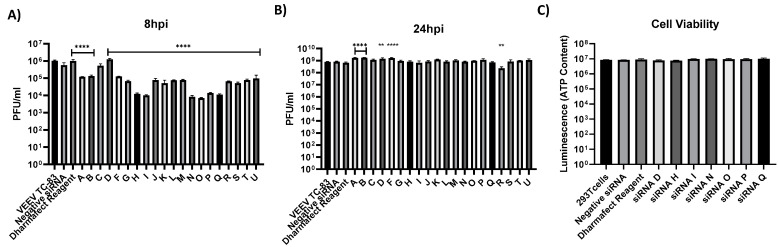
Anti-VEEV siRNAs can cause reduction in VEEV TC-83 replication. (**A**,**B**) 293T cells were transfected with 20 nM individual VEEV specific siRNAs 24 h prior to infection. Transfected cells were then infected with VEEV TrD at an MOI of 0.1 and supernatants were collected at 8 hpi (**A**) and 24 hpi (**B**). Viral titers were determined via plaque assay. (**C**) Cytotoxicity of siRNA was determined in 293T cells using CellTiter-Glo Luminescent Cell Viability Assay 24 h post transfection. Negative siRNA is nonspecific to VEEV and human genome. Graphs represent data from two independent experiments performed in triplicates (*n* = 6). ** *p* < 0.0021, **** *p* < 0.0001 compared to negative control siRNA.

We quantified the virus multiplication inhibitory potential of the vsiRNAs by transfecting them into 293T cells followed by quantification of viral load by plaque assays. 293T cells were plated in 96-well plates 24 h prior to transfection. The cells were transfected with the vsiRNAs (20 nM), indicated in Table 1, 24 h prior to infection by TC-83 (MOI: 0.1, 0.01). Untransfected, TC-83 infected cells, cells transfected with a nonspecific siRNA and infected with TC-83, cells treated with the transfection reagent alone and infected with TC-83 were maintained alongside as positive and negative controls. Culture supernatants were obtained at 8 and 24 h post infection (hpi) and viral load was quantified by plaque assay. At the 8 hpi timepoint, the vsiRNAs A, B, F, G, J, K, L, M, R, S, T and U demonstrated one log reduction in infectious viral titer as compared to the VEEV TC-83 positive control (Figure 1A). VsiRNAs C and D did not demonstrate any significant reduction in infectious viral titer as compared to the VEEV TC-83 control. The vsiRNAs H, I, N, O, P and Q demonstrated statistically significant, two log reduction in the infectious titer when compared to the positive control. The fold changes in viral titers as achieved by these vsiRNAs at the 8 h time point, when compared to the positive control are indicated in Figure 1B. The supernatants analyzed at the 24 h time point did not demonstrate statistically significant reduction in infectious titer and the viral loads in the vsiRNA-transfected cells were comparable the untransfected, TC-83 infected control. This suggested that the inhibition achieved by the vsiRNAs was short-lived and the virus was able to overcome the inhibition. No apparent cytotoxicity related to the vsiRNAs was observed as determined by CellTiter Glo assay at 24 h post transfection (Figure 1C).

### 3.3. Impact of Efficient vsiRNAs on Viral Multiplication Kinetics

**Figure 2:**
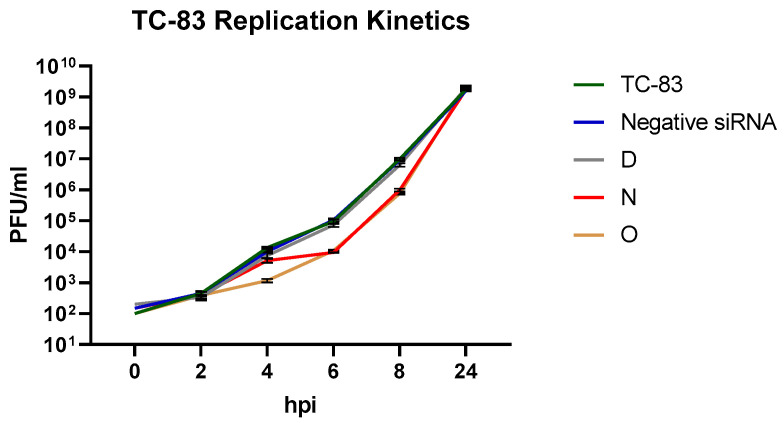
Twenty-four hours prior to infection, 293T cells were transfected with 20 nM anti-VEEV siRNA D, N, O and negative siRNA. Cells were infected with TC-83 at MOI 0.1 and supernatants were collected at 0, 2, 4, 6, 8 and 24 hpi. Viral titer was determined via plaque assay. Graphs represent data from two independent experiments (*n* = 6).

We queried the impact of vsiRNAs that demonstrated robust inhibition at the 8 h time point on the viral multiplication kinetics to determine the effect on early and late stages of the 24 h infection period. VsiRNAs N and O were considered, with vsiRNA D as a comparison control for a candidate that was incapable of achieving inhibition. The untransfected, TC-83 infected sample and the nonspecific siRNA infected samples were maintained as positive and negative controls. VsiRNA O demonstrated slower replication than the reference controls up to 4 hpi (Figure 2). The replication kinetics for both vsiRNAs N and O increased steadily, however, and reached the same titers as the reference controls by the 24 h timepoint.

### 3.4. Efficacy of vsiRNAs in Reducing TrD Multiplication

**Figure 3:**
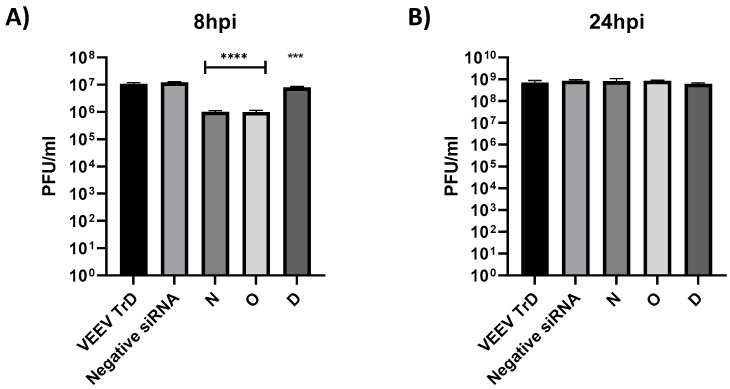
Antiviral activity of selected anti-VEEV siRNA against VEEV-TrD. (**A**,**B**) 293T cells were transfected with 20 nM individual VEEV specific siRNAs 24 h prior to infection. Transfected cells were then infected with VEEV TrD at an MOI of 0.1 and supernatants were collected at 8 hpi (**A**) and 24 hpi (**B**). Viral titers were determined via plaque assay. Graphs are representative of one independent experiment (*n* = 3). *** *p* < 0.0002, **** *p* < 0.0001 compared to negative control siRNA.

The efficacy of vsiRNAs N and O in inhibiting TrD multiplication was determined following the same procedure as outlined for TC-83. 293T cells were transfected with either of the vsiRNAs 24 h prior to infection (MOI: 0.1). The supernatants were harvested at 8 and 24 hpi and infectious titer was quantified by plaque assay. Data presented in Figure 3 recapitulate the observations made with TC-83, in that inhibition can be observed at the earlier time point (8 hpi (Figure 3A)), which was overcome at the later time point (24 hpi (Figure 3B)). The extent of inhibition observed at the 8 h time point in the context of TrD infection appears to be slightly lower for vsiRNAs N and O (1 log) as compared to that observed with TC-83.

### 3.5. Dose Dependence and Impact of Combination of siRNA on Antiviral Efficacy

**Figure 4:**
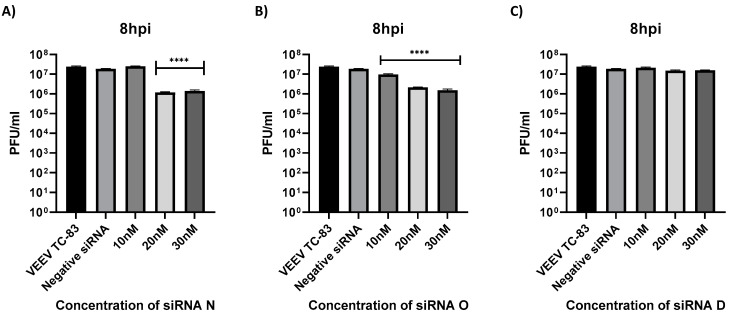
Dose dependent impact of VEEV siRNA. (**A**–**C**) 293T cells were transfected with either 10, 20 or 30 nM of siRNA. At 24 h post transfection, cells were infected with VEEV TC-83 at an MOI 0.1 and supernatants were collected at 8 hpi. Viral titers were determined via plaque assay. Graphs are representative of one independent experiment (*n* = 3). **** *p* < 0.0001 compared to negative control siRNA.

Dose dependency of inhibition on vsiRNA concentration was determined in the context of TC-83 by assessing inhibition at the 8 h time point following transfection by increasing concentrations of the vsiRNAs N and O (10, 20 and 30 nM). 293T cells were transfected with the vsiRNAs, or vsiRNA D, which did not demonstrate inhibition 24 h prior to infection with TC-83 (MOI: 0.1). Untransfected, TC-83 infected cells were maintained as positive controls. Supernatants were quantified at 8 hpi by plaque assay. Data provided in Figure 4 indicate that at the lowest concentration tested (10 nM), none of the vsiRNAs exhibited antiviral activity. At the 20 nM and the 30 nM concentrations, both N and O vsiRNAs demonstrated inhibition. The extent of inhibition plateaued at 20 nM concentration and no statistically significant change in inhibition was observed at 30 nM concentration of the two vsiRNAs. 

**Figure 5:**
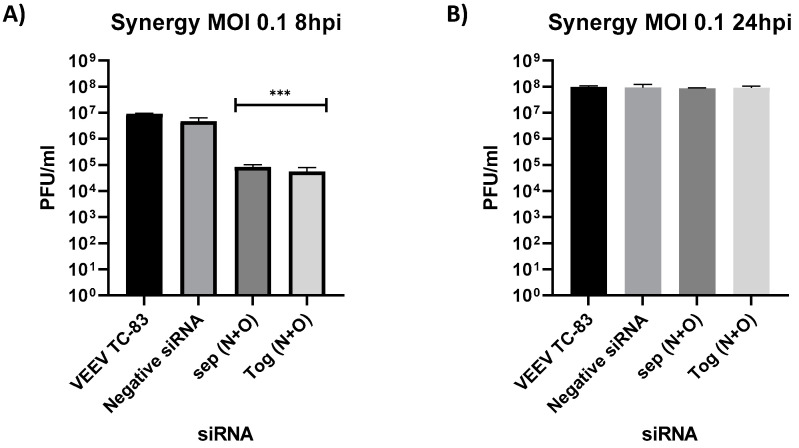
Impact of siRNA combinations on antiviral efficacy. (**A**,**B**) 293T cells transfected with siRNA N and siRNA O either by mixing them together (N + O) or by separate (N + O). (**A**) Both siRNAs were mixed in the same tube with the DharmaFECT1 reagent and then added dropwise on the cells. (**B**) Both siRNAs were mixed in separate tubes with DharmaFECT1 reagent and then added dropwise on the cells. At 24 h post transfection, cells were infected with VEEV TC-83 at MOI of 0.1 and supernatants were collected at 8 and 24 hpi. Viral titers were determined via plaque assay. Graphs are representative of two independent experiment (*n* = 6). *** *p* < 0.0002 compared to negative control siRNA.

The impact of combining the two effective vsiRNAs on the inhibitory potential was next determined in the context of TC-83 infection. The vsiRNAs N and O were combined at 20 nM concentration each into a single tube and transfected into 293T cells (N + O). At 24 h post transfection, the cells were infected with TC-83 (MOI: 0.1). In addition, the impact of transfecting the two vsiRNAs as concomitant, individual transfections were assessed (N) + (O). Supernatants were quantified for infectious virus titer at 8 and 24 hpi. Data presented in Figure 5 indicate that both experimental scenarios yielded inhibition at the 8 h time point when compared to the comparison controls, and the inhibition was overcome at the 24 h time point. 

### 3.6. Mechanistic Assessment of Antiviral Activity of vsiRNAs

**Figure 6:**
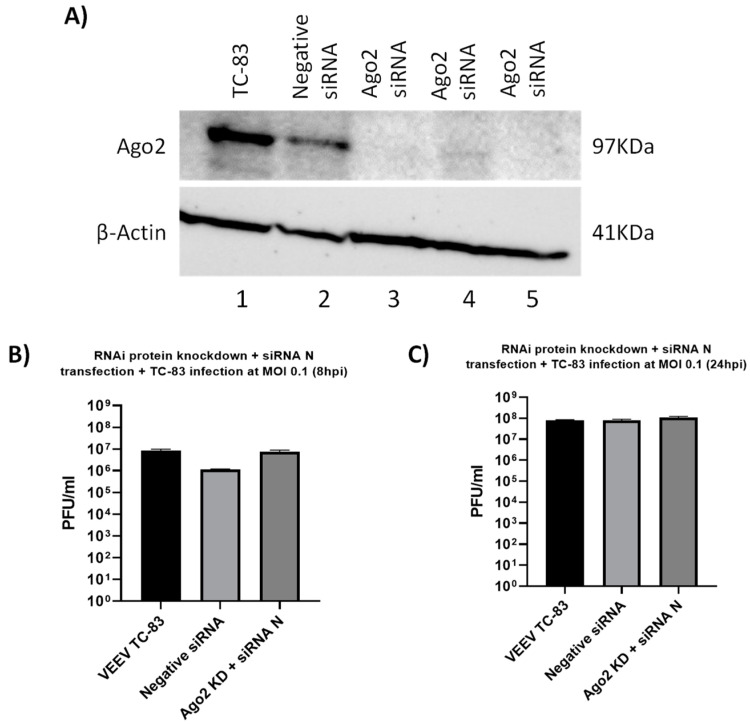
Mechanistic assessment of antiviral activity of vsiRNA. (**A**–**C**) 293T cells were transfected with 20 nM anti-human Ago2 siRNA for 72 h. Ago2 knockdown cells were then transfected with siRNA N for 24 h prior to infection with VEEV TC-83 at an MOI of 0.1. (**A**) Cell lysates were analyzed by Western blot and probed for Ago2 to determine knockdown and actin as a loading control. (**B**,**C**) Culture supernatants were collected at 8 and 24 hpi and viral titer was determined via plaque assay. Graphs are representative of one independent experiment (*n* = 3).

Argonaute 2 protein (Ago2) is a critical member of the RNAi pathway and plays a central role in the RNAi-mediated restriction of gene expression. To ascertain that the mechanism of inhibition by the effective vsiRNAs involved the RNAi pathway, Ago2 was depleted by transfection of anti-Ago2 siRNAs into 293T cells. Specifically, 293T cells were transfected with 20 nM of human *Ago2* siRNA and 72 h post transfection, the vsiRNA N was transfected into the cells. At 24 h post transfection of the vsiRNA, the cells were infected with TC-83. Samples for Western blot analysis were obtained at 24 hpi. The depletion of Ago2 in triplicate samples was verified by Western blot analysis (Figure 6A, lanes 3–5). The impact of Ago2 depletion on vsiRNA N-mediated inhibition of TC-83 was assessed at 8 hpi. The rationale behind this experiment was that if the RNAi pathway was indeed the mechanism behind the antiviral activity, depletion of Ago2 will render the pathway ineffective, thereby eliminating the antiviral outcome. Data included in Figure 6B support this rationale as the inhibition by vsiRNA N was lost at the 8 h time point. 

### 3.7. Interaction of VEEV nsP2 with the RNAi Pathway

**Figure 7:**
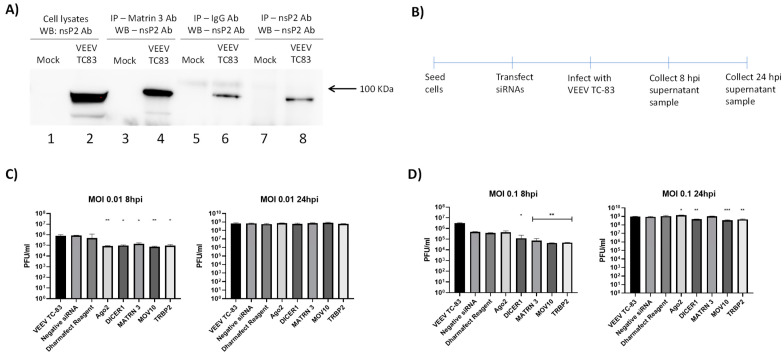
VEEV interaction with RNAi pathway. (**A**) 293T cells were infected with VEEV TC-83 at an MOI of 1.0 and lysates were collected at 18 hpi. Lysates were immunoprecipitated using anti-Martin 3, anti-nsP2 and IgG antibodies, and analyzed by Western blot to determine interaction with nsP2. (**B**) Schematic representation of steps involved to knockdown RNAi pathway protein. (**C**,**D**) 293T cells were transfected with siRNAs for Ago2, Dicer, MOV10, TRBP2 or Martin 3. (**C**) Cells were infected with TC-83 at an MOI of 0.01 and cell supernatants were collected at 8 and 24 hpi. (**D**) Cells were infected with VEEV TC-83 at an MOI of 0.1 and cell supernatants were collected at 8 and 24 hpi. Viral titer was determined via plaque assay and negative siRNA was used as a control. Results are representative of three independent experiments (*n* = 3). * *p* < 0.0332, ** *p* < 0.0021, *** *p* < 0.0002.

The observation that the vsiRNA-mediated depletion was observed at the earlier time point post infection (8 hpi) but overcome at the later time point (24 hpi) prompted us to query if there was any interaction of VEEV with the RNAi machinery. Specifically, we were interested in nsP2 because both N and O vsiRNAs target the nsP2 region of the VEEV genome. 293T cells were infected with TC-83 (MOI: 1) and 16 hpi, total protein lysates were generated. The lysates were subjected to immunoprecipitation using a commercially available nsP2 antibody. Immunoprecipitated samples were analyzed using liquid chromatography tandem mass spectrometry (LC–MS/MS) to identify potential host cell proteins that interact with VEEV nsP2. Background subtraction was conducted with mock-infected control samples to eliminate nonspecific interactions. Results obtained from three independent MS runs showed that Matrin3 protein interacted with nsP2. The interaction of Matrin with nsP2 was independently validated by immunoprecipitation and Western blot analysis (Figure 7A). We hypothesized that the interaction of the Matrin3 protein with nsP2 suggests that the RNAi pathway may be an important innate immune mechanism that can impact VEEV multiplication in infected cells. To ascertain if the components of the RNAi machinery can influence VEEV replication, siRNA-mediated depletion was carried out for specific target proteins in the siRNA pathway, namely, Dicer, MOV10, TRBP2 and Matrin 3, after which the cells were infected with TC-83 (MOI: 0.1, 0.01). The schematic is illustrated in Figure 7B. The ability of these siRNAs to successfully deplete their target proteins was confirmed and the data are included in Supplemental Materials Figure S1. Supernatants were collected at 8 hpi and infectious titer quantified by plaque assay. Data included in Figure 7C indicate that at the lower MOI, the depletion of all the above-mentioned RNAi pathway targets decreased viral load in a statistically significant manner. At the higher MOI (Figure 7D), depletion of all targets except Ago2 impacted viral load. Cumulatively, these observations suggest that the RNAi machinery is an important innate immune component that influences VEEV infection.

## 4. Discussion

The RNAi pathway is an evolutionarily conserved innate immune response that plays an important role in host–pathogen interactions as evidenced in the context of several acutely infectious viral agents [17]. Our initial objective in this study was to design vsiRNAs that can target the VEEV genome and use that strategy to develop novel solutions to treat encephalitic alphavirus infection. Such an approach has been effectively employed before and four siRNAs were demonstrated to successfully decrease VEEV viral load in cell culture [23]. Encapsulated siRNAs that target viral genomes have also demonstrated efficacy in the context of nonhuman primates and conferred protection after exposure to Ebola Virus [24,25,26]. In the current study, our goal was to increase the repertoire of vsiRNAs that can successfully deplete the virus in infected cells and potentially expand into in vivo applications. The 20 newly designed vsiRNAs targeted conserved regions of both TC-83 and TrD strains of VEEV (Table 1). Among the 20v siRNAs evaluated, vsiRNA H (nsP3), I (nsP2), M (nsP3), N (nsP2), O (nsP2), P (nsP2) and Q (nsP4) reduced TC-83 viral titer by 100-fold while vsiRNAs C (E1) and D (nsP2) were ineffective (Figure 1). 

Interestingly, we observed that this antiviral effect was overcome at the 24 h time point and the inhibition was not sustained. This may be explained by a few possibilities: (1) The effective intracellular concentration of the vsiRNAs was sufficient to bind to the respective targets when the genomic copy numbers were low during the earlier time points. This possibility is supported by data presented in Figure 2. As the number of genomic copies increased, the effective concentration of the vsiRNAs was insufficient to engage all available genomic targets, thus leading to increase in viral load. (2) The stability of the transfected vsiRNAs decreased over time, which could also further contribute to the intracellular effective concentration. (3) VEEV may develop resistance to the vsiRNAs by an as-yet unknown mechanism by possibly suppressing the RNAi pathway. Resistance may be conceptually possible due to generation of escape mutants that have altered nucleotide sequence at the target sites of siRNA. However, prior research has demonstrated that the mechanism of siRNA resistance in VEEV TrD strain is not due to escape mutants as mutations did not readily arise at the conserved sequences [23]. The study did not identify any silent mutations in the siRNA target sequences in the resistant viruses that may have contributed to the resistance. (4) It may be possible that virus-encoded proteins may interact with the RNAi pathway to suppress its antiviral innate immune function. Many mammalian viruses encode proteins that function as suppressors of RNA silencing, such as nucleocapsid protein of coronavirus [27], capsid of yellow fever virus [28], VP55 of vaccinia virus [19,20] or capsid of Zika virus [29]. Mass spectrometry analysis of VEEV nsP2 interaction partners has identified the Matrin3 protein of the RNAi machinery as immunoprecipitating with nsP2, which was independently verified by immunoprecipitation (Figure 7A). It remains to be determined if there is a direct interaction between Matrin3 and nsP2. However, the observation that depletion of the RNAi machinery components decreased viral load (Figure 7B) is inconsistent with the idea of suppression of the RNAi pathway by nsP2. Thus, the interaction between VEEV nsP2 and the RNAi pathway, while being an interesting and novel observation, requires additional investigation to assess proviral or antiviral outcomes. An interesting line of inquiry could be if these proteins are subject to proteolytic cleavage by nsP2. VEEV nsP2 has been demonstrated to cleave host proteins with innate immune antiviral functionality, such as TRIM14 [30].

Potential additive inhibitory outcomes when combining siRNAs can potentially provide combinatorial intervention strategies. In the current series of experiments however, combining vsiRNAs N and O did not result in an additive inhibitory effect (Figure 5). The binding sites of the two vsiRNAs are separated on the viral genome, thus making it possible that a single genome may be bound by both vsiRNAs, and therefore not providing an additive effect over the binding of a single vsiRNA. If the target sites for the two vsiRNAs overlapped, it may be possible that they may bind competitively, thus allowing additional genomes to be targeted to achieve greater inhibition than the individual vsiRNAs. In the context of CHIKV, simultaneously silencing multiple genes using a combination of siRNAs enhanced the antiviral activity and was found to be a better strategy for preventing CHIKV replication in both in vitro and in vivo studies [31,32]. Ongoing studies in our laboratory are focused on combining more than two vsiRNAs as an encapsulation strategy to address increased inhibition.

## 5. Conclusions

The current study supports the possibility of vsiRNA to function as an effective antiviral strategy to inhibit infectious viral titers in cell culture and in animal models. While this proof-of-concept study provides support to the approach, further development of the strategy to deliver effective countermeasure solutions will require rational combination of vsiRNAs that can target various regions of the viral genome, which can not only enhance the extent of inhibition, but also decrease the potential for emergence of suppressive mutations in the viral genome. Optimization of delivery mechanisms such as encapsulation can add to the countermeasure development roadmap by conferring increased stability in vivo and enhancing bioavailability in hard-to-reach target tissues such as the brain.

## Figures and Tables

**Table 1:** vsiRNA sequence and genomic location of target.

siRNA	vsiRNA Sequence (Sense Strand)	Genomic Location	Gene Target
**A**	GGUCAAAGGUGCAGCUAAA	4202–4220	nsP3
**B**	GGAAAUAACUGAUAAGGAA	359–377	nsP1
**C**	GAGCAAUGGAAGAAAGAUA	10721–10739	E1
**D**	AGGAAUAAUCACUGGGAUA	3276–3294	nsP2
**F**	UGGGAAACGUUUAAAGAAA	6411–6429	nsP4
**G**	UGGAAUGCUUCAAGAAAUA	6373–6391	nsP4
**H**	GGAAAUGACUCUCAAGGAA	4475–4493	nsP3
**I**	UCGCCGUUGCACUAAAUCU	2576–2594	nsP2
**J**	CCAGGAAGGUGGAGAACAU	5899–5917	nsP4
**K**	GAACUUGGCUGGAGCAUAU	602–620	nsP1
**L**	UGAACAAGUCAUAGUGAUA	1784–1802	nsP2
**M**	GAAGAAGAGGAUAGCAUAA	5160–5178	nsP3
**N**	GCGAAUACCUGUACGACAU	2038–2056	nsP2
**O**	AGACUAAGAUUGUGAUUGA	2656–2674	nsP2
**P**	GGUACAAGGUGAAUGAAAA	2827–2845	nsP2
**Q**	GAAAGUGACUCCAGGAACA	6581–6599	nsP4
**R**	CGUUAAUGAUUCUGGAAGA	6859–6877	nsP4
**S**	CGGCGAAAUUUCAUCAAUA	6923–6941	nsP4
**T**	CGAAGAAAGCAUCCAAAUA	8061–8079	Capsid
**U**	GGAGAAAGCAUGAGCAGUA	4752–4770	nsP3

## Data Availability

Not applicable.

## Supplementary Materials

The following supporting information can be downloaded at: https://www.mdpi.com/article/10.3390/v14081628/s1, Figure S1: Knock down of RNAi pathway proteins.
