# Supplementary figures and images for: Inhibition of Venezuelan Equine Encephalitis Virus Using Small Interfering RNAs
Source: Viruses. 2022 Jul 26;14(8):1628. doi: 10.3390/v14081628 (PMC9331859; doi:10.3390/v14081628)

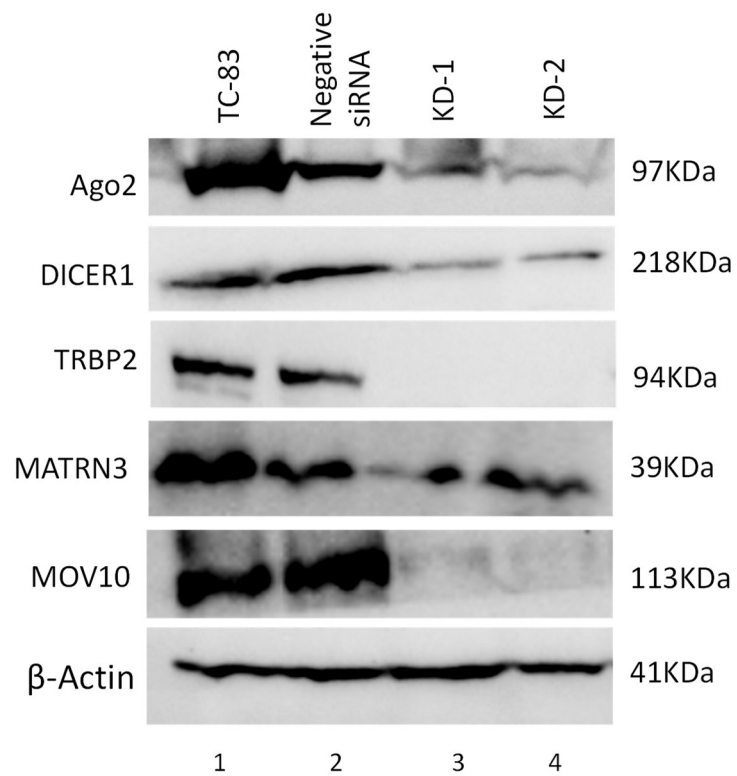

**Figure S1.** Knock down of RNAi pathway proteins.

Supplement: Supplementary file 1 [file viruses-14-01628-s001.zip › viruses-1794458-supplementary.pdf]
